# Hospitals—Voluntary or Self-Supporting?
*An address delivered in Melbourne to a lay audience after consultation with the President and Council of the Victorian Branch of the British Medical Association.


**Published:** 1930

**Authors:** R. J. A. Berry

**Affiliations:** Medical Director, Stoke Park Colony, Stapleton, Bristol; formerly Dean of the Faculty of Medicine and Professor of Anatomy in the University of Melbourne


### HOSPITALS—
### VOLUNTARY OR SELF-SUPPORTING ? *

BY

R. J. A. Berry, M.D., F.R.C.S., F.R.S. Edin.,

*Medical Director, Stoke Park Colony, Stapleton, Bristol ; formerly Dean of the Faculty of Medicine and Professor of Anatomy in the University of Melbourne.*

---

In 1927, as the accredited representative of the State of Victoria and the University of Melbourne, I travelled some 30,000 miles on a special mission of inquiry into Hospital and Medical problems. As a result of this inquiry it became clear that the world's practice to-day is to concentrate all medical effort—hospitals, laboratories and pre-clinical sciences—on the one spot, and to endeavour to bring all medical knowledge to bear on the study, treatment, and prevention of disease. This new procedure has resulted in the creation of new types of hospitals, and has been responsible for a large amount of new medical and hospital building and administration. This newer form of medical policy may be termed the " Medical centre devoted to community health, and its associated hospital, the self-supporting hospital," whilst the older type, with which most of us are familiar, is the dispersed and scattered medical school and laboratory with the associated " voluntary hospital."

* An address delivered in Melbourne to a lay audience after consultation with the President and Council of the Victorian Branch of the British Medical Association.

The world's best modern medical practice is to-day based on a tripod—a tripod of medical education, research and administration—and the whole is entirely directed to the patient. The patient should be treated as an individual, not as a separate series of specialities, hence the necessity to-day of concentration of medical effort, and not its dissipation in non-correlated hospitals and laboratories, as in Melbourne and Bristol at the moment. The complete medical school is, therefore, the first essential; and the second is the closest association of that complete medical school with a hospital or group of hospitals. The hospital is thus the second, not the first, necessity of the medical centre devoted to community health, for it is the obvious duty of modern medicine to keep people out of hospitals rather than in them. Two corollaries follow: (1) That the hospitals of the medical centre should be open to all classes of patients, irrespective of their poverty or means; and (2) that the association of the word "hospital" with the erroneous modern democratic notion of getting something for nothing—free treatment by the medical profession—should be abandoned.

Great Britain is the home and origin of the voluntary hospital, and as first introduced it was simply a charity designed for the indigent poor. By "voluntary" was meant free service by the medical profession; free administrative service by the lay and business communities; free and voluntary financial contributions on the part of the public, and voluntary attendance by the poor and sick. The whole was originally a noble charity. It is, therefore, no wonder that there are still those who are loath, in defiance of all evidence to the contrary, to desire any change in a scheme which has done such magnificent service for rather more than a century. On the other hand, it is surely obvious that the future of this voluntary principle, as applied to hospitals and medical service to the community, is fraught with difficulties and national dangers, and to-day there are many in all parts of the world who see both the drift and the precipice confronting the voluntary hospital.

Originally, and up to the beginning of the present century, the British voluntary hospital did fully meet the needs of the times, and it compared favourably with the hospital system of any other country. To-day English expert opinion urges against it, at least in so far as its medical services to the nation are concerned, that the voluntary hospital system (1) has not developed in proportion to the needs of the community; (2) has failed to meet the growing needs of the middle classes; (3) has largely failed to maintain an adequate standard in regard to sites and buildings, and has not recognized the absolute necessity of the associated pre-clinical laboratory in the study and treatment of disease; and (4) has failed to co-ordinate its hospital services in the interests of the community, and must continue to do so so long as its several units remain dissociated, non-correlated, and merely mutually competitive.

The present generation has become so accustomed to the prevalent and perverted notion that something can be had for nothing—education, medical attendance, protective doles from the State, and the like—that it forgets, if it ever knew, that free medical treatment in voluntary hospitals is the growth and practice of little more than a century, and that such free medical treatment was then, and is now, only given on the distinct understanding that the recipients of this medical charity are really the poor and indigent sick.

But this is very far from being the case to-day, and there is probably not one so-called voluntary hospital of first-class standing in the British Empire which has not been compelled to depart from the main principles of voluntaryism. Voluntary hospitals are now compelled to demand payment from their patients for board, lodging and food, and for financial reasons associated with their own balance sheets admit many others than the indigent sick. But the third great principle of voluntaryism—upon which all depends—free medical service by the profession is still retained in its original integrity.

During the last twenty-five years, and especially in the last fourteen of these, there have been two main factors at work, insidiously, slowly, but surely, tending to destroy the voluntary principle of hospitals: (1) the rapid progress of medicine, intensified by the war and its problems, for medicine learnt as much from the war as did aviation; and (2) that war itself—the greatest and most destructive in history. The former has compelled all hospitals to extend their medical activities, to embark upon many new departments, to expand old ones, to handle an ever-increasing number of patients, to install most expensive therapeutic plants, with the result that their expenditure has doubled and trebled. The latter, on the other hand, has simultaneously impoverished the peoples of Great and Greater Britain, and has thus largely impaired the sources of hospital revenue.

These facts can be shown in two very striking ways. Between the years 1913 and 1925 the cost of upkeep per bed in the London voluntary hospitals has increased by 100 per cent., and of this present-day cost of £200 per bed per annum—it is more in Melbourne—some £93 has to be found by piteous charitable appeal—an appeal made to the most heavily-taxed people in the world, staggering under a load of debt and taxation.

Further, there have been added to London's hospitals since the war some 1,600 additional beds. It is difficult to understand how the voluntary system of charitable doles to voluntary hospitals, largely and increasingly unable to meet the present requirements of either patient or advancing medical science, is to cope with this new burden. Many patients who formerly would not only have sought and paid for private medical advice, but would have themselves contributed their charitable offerings to the voluntary hospital and the indigent sick, now do neither one nor the other, but themselves seek free hospital medical treatment, partly from economic reasons, and partly because there is a growing feeling that the well-equipped hospital can give better service than the private practitioner or the private nursing home.*

It is, therefore, no wonder that Dr. Joseph Griffiths, of Cambridge, writes in 1927 that "I believe it would be better if all were now to open their eyes to see the new movement which is rapidly replacing the old voluntary or charity systems, with their serious limitations." Or that the *Westminster Gazette* can say: "Unless the voluntary hospitals can revolutionize their finance, they are doomed." Or that Prince Arthur of Connaught tells us that "the popular impression that the large voluntary hospitals are merely charitable institutions must be replaced by a consciousness that they are the guardians of the nation's health." If this be so—and there are few experts who would deny it—then it is clearly a national

* Contributory schemes which now exist in Bristol and other cities may make these remarks inapplicable in every locality, but such schemes are the exception, not the rule.—EDITOR.

blunder to expect them to function apart from the medical centre devoted to community health, or to be financed by public and insufficient charity and the voluntary and gratuitous services of the most eminent surgeons and physicians, or to continue longer to believe that medical skill—one of the most expensive things in the world to acquire—can still be had for nothing.

Educated as I was under the British voluntary system in the height of its prosperity and strongly prejudiced in its favour, it is, nevertheless, impossible to pass, as I did in 1927, from an examination of the voluntary hospital system of Britain to the self-supporting types of Canada and America without being profoundly impressed with the latter.

In all the great Canadian and American hospitals visited by me totally new hospital principles have been put into operation in order to ensure that the patient shall have the best that medicine has to offer. First, there is hardly anywhere a general hospital, of the type of those with which we are familiar in Great Britain and Australia, totally divorced from the pre-clinical and scientific laboratories of a medical school. The whole staff, university, laboratory, pre-clinical and clinical, is concentrated on the one spot, on the group of associated hospitals, and thus every patient in those hospitals may benefit by the collective skill of the whole, rather than only the part as in Melbourne. Second, these Canadian and American Class "A" university hospitals are open to all, irrespective of the social or financial status of the individual, and it is exactly here that these Canadians and Americans have so boldly departed from the older British voluntary system confined to the indigent sick.

It is to-day the clear and obvious duty of our large medical centre hospitals to give every type of patient free access to their medical facilities and beds, for only there, where all medical effort is, or should be, concentrated, is it possible for medicine to give of its best; and all patients are entitled to that best, and should not be asked or expected to simulate poverty in order to get it. But the labourer is worthy of his hire, and it is illogical in the extreme, nay, it is grossly unfair, to suppose and assume that, because the British type of voluntary hospital was originally based on voluntary and gratuitous service by all concerned, that this more modern and totally different duty of medicine can be maintained on the same basis, that is, free service by the medical profession. In other words, if all classes of patients are to be admitted to our great medical centre Class "A" hospitals, then the medical staffs of those hospitals must be paid for their services. In every one of the great Canadian and American hospitals visited by me in 1927 I found every member of the staffs of both medical school and hospitals, pre-clinical and clinical, paid and well paid for their hospital services, and a contented staff means efficient service. The educated Canadian and American has at least learnt the futility of constantly seeking something for nothing.

Mr. A. H. Leaney, of Birmingham, a non-medical manager of the general hospital in that city, who made like myself a special study of the Canadian-American self-supporting hospital, sums up his impressions as follows:—

1. In the self-supporting hospital all classes receive equally good care.

2. All have the benefit of the resources of a modern hospital.

3. All classes become acquainted with the work of the hospital and give it moral and financial support.

4. The unsatisfactory system of private nursing homes is replaced.

It is quite certain that if universities, hospitals and medicine generally are to do their duty to the community, medical schools and hospitals must have adequate financial support, and this cannot be much longer maintained under the present system of inadequate financial Treasury grants and piteous appeals to the public. In the Canadian-American self-supporting hospitals the sources of revenue are :—

1. Receipts from endowments. No wealthy man dare die in the States without leaving something to his university, home town or favourite hospital.

2. Payments for medical and other hospital services from middle class and wealthy patients, from both of whom, but particularly the latter, certain profits accrue, just as they do in any other business or profession.

3. Payments from province, state, city or municipality for the actual costs of board, lodging and medical treatment accorded those of their people who, unable to pay themselves, still seek hospital treatment.

In all these self-supporting hospitals accurate "costing" accounts are kept of every patient. There is a complete register of the actual costs of his food, board, lodging, medical and specialist fees and the like. In the cases of the paying patients the patient himself discharges the bill, and as it includes his medical fees, these are paid over to the medical consultant in charge of the case. For the indigent sick, unable to foot the bill, that bill is presented to the city, borough or municipality whence he came, and is discharged by them. Under this system the medical profession and everybody working in the hospital receives payment for services rendered. The hospitals are not in debt; there are no piteous appeals for charity; there is no inferiority complex on the part of any paying patient attending the hospital; there is no pauperization of the people, and there is no exploitation of the medical profession, of which last there has, perhaps, been in the past a little overmuch.

There can surely be few who do not view the future of our voluntary hospitals with both apprehension and misgiving, and there can be none, at least in the medical profession, who do not realize that to-day concentration of medical effort is the key to the position, especially in a university town. Bristol is indeed fortunate in having a medical school in its midst, but it seems just a little doubtful if the necessity for radical reconstruction of schools and hospitals on the one spot, as has been so ably pleaded by the Professor of Surgery in this University, has yet received the serious consideration which is its due.

The medical visitor to Winnipeg, Toronto, Montreal, or any of the first-class American universities will be astounded to see that everywhere, since 1920, there has been a wholesale reconstruction of hospitals and university medical schools, and always with the same objective—the concentration of all medical effort, science and skill on the one spot, for the good of the patient and the better education of the medical man. Nor are these North Americans alone in their views as to the necessity to-day for the unification of both clinical and pre-clinical sciences in the only place where they can be really effective, that is the hospitals and schools of the medical centre, for Birmingham and Aberdeen have now both taken the preliminary steps for such a reconstruction.

By a curious coincidence it was my pleasurable task, in connection with the report which I had recently to make to the Victorian Government on this very problem, to visit both Bristol and Cardiff, for the study of their respective methods of handling these modern teaching hospital and university problems. The conclusion was formed that, though Bristol had yet a long way to go before attaining the modern ideal, it was at least an excellent position city for such a concentration of medical school and hospital. The Centenary of the Bristol Medical School approaches. Could there possibly be any better way of celebrating such a noteworthy occasion than by leading the van of English medical progress, and fusing our great hospitals and Medical School into one even greater Bristol University Medical Centre ?

